# Synergistic Enhancement of Humoral and Cellular Immunity in Attenuated Live Vaccines Using Chemical Nanoadjuvants: Evaluation of Sustained-Release Formulations

**DOI:** 10.3390/vetsci12090899

**Published:** 2025-09-17

**Authors:** Jianli Shi, Chang Liu, Chen Li, Jun Li

**Affiliations:** Key Laboratory of Livestock and Poultry Multi-Omics of Agriculture and Reral Affairs, Shandong Research Center of Livestock and Poultry Biologicals Engineering, Institute of Animal Science and Veterinary Medicine Shandong Academy of Agricultural Sciences, Jinan 250100, Chinaliuchangsaas@163.com (C.L.)

**Keywords:** CSFV vaccines, adjuvants, CpG, sustained-release agents

## Abstract

Achieving a balance between enhancing both humoral and cellular immunity while ensuring sustained release for attenuated live vaccines has long been a challenge in the vaccine industry. However, the incorporation of chemical materials as nanoadjuvants has emerged as a promising strategy to address this challenge. In this study, a novel nano-adjuvant with sustained release properties was developed by combining polyethylene glycol (PEG) and other biochemical and molecular biology agents with CpG. The efficacy of this adjuvant was assessed using the widely applied CSF attenuated live vaccine. The body temperature and relative daily weight gain results demonstrated the safety of the formulations. Importantly, the combination of CpG immune enhancers and sustained-release agents led to a synergistic augmentation of both humoral and cellular immune responses. Specifically, the combination of PEG20000 and benzoic acid, along with Seppic white oil, significantly enhanced humoral immunity. In contrast, the combination of total white oil, PEG6000 and benzoic acid demonstrated superior efficacy in promoting cellular immunity. This study offers important insights into enhancing vaccine efficacy through novel adjuvant combinations, which can be applied to the development of more effective vaccines in the future.

## Simple Summary

A novel nano-adjuvant with sustained release properties was developed by combining polyethylene glycol (PEG) and other biochemical and molecular biology agents with CpG. The combination of PEG20000 and benzoic acid, along with Seppic white oil, significantly enhanced humoral immunity. In contrast, the combination of total white oil, PEG6000 and benzoic acid demonstrated superior efficacy in promoting cellular immunity. These findings underscore the differential roles of sustained-release agents in shaping immune outcomes and provide a strategic framework for optimizing attenuated live vaccine formulations through adjuvant synergy.

## 1. Introduction

Classical swine fever (CSF), caused by CSF virus (CSFV), is one of the most devastating viral epizootic diseases of swine in many countries, listed as a Class A animal disease by the World Organization for Animal Health (WOAH) [1]. CSFV is often a coinfection with other pathogens such as porcine reproductive and respiratory syndrome virus (PRRSV), porcine circovirus type 2 (PCV2) and pseudorabies virus (PRV). There is no effective drug treatment after CSFV infection, which can only be prevented and protected by vaccine [2,3].

By mingling with the antigen components in the vaccine, adjuvants can enhance the immune response of the vaccine, especially for antigens with insufficient immunogenicity, enabling them to produce faster and better immune responses [4]. Among them, CpG motifs have the advantage of being able to stimulate endogenous immune responses more quickly, characterized by the production of multiple Interleukins and use as an immune adjuvant [5,6]. CpG has shown a good application prospect in the field of immunoenhancers, and has become a research hotspot to treat animal diseases and even human diseases in recent years [7]. CpG adjuvant (CpG 1018) has been developed as a vaccine adjuvant in the Dynavax’s hepatitis B vaccine and COVID-19 vaccine for the emergency scope of application. Our groups have also confirmed that the CpG motif could enhance the immune effect of the PCV2 DNA vaccine [8].

Sustained-release agents are a class of substances that can slowly release and exert long-term effects after wrapping vaccines. The development of sustained-release agents for vaccines has become an expanding field for generating stronger vaccines effects in the last thirty years [9]. Biochemical and molecular biology preparations used as immunological adjuvants and sustained-release agents can help to extend the duration vaccine affect. It is reported that many compounds such as white oil and benzoic acid also have sustained-release effect [10,11]. In this work, CpG and five different sustained-release agents were emulsified and immunized with the CSF vaccine to evaluate the immune efficacy.

## 2. Materials and Methods

### 2.1. Animals, and Vaccine

Seventy 35-day-old commercial Landrace piglets tested negative for CSFV antibody from Shandong Zhongba Agriculture and Animal Husbandry Development Co., Ltd. were selected for vaccine and randomly divided into seven groups (A–G), ten piglets in each group, and housed separately. All experimental animals were euthanized after the experiment. The method of euthanasia for experimental animals is electrocution and exsanguination as a secondary step within 15 s of initial stunning. CSF live vaccine was bought from Luoyang Huizhong Biology (No. 210512) and used according to the protocol supplied by the manufacturer with 2 doses per pig of all groups.

### 2.2. Experimental Design

The required CpG doses and sustained-release agent were emulsified according to the number of immunized pigs. In order to evaluate the effect of storage time, the emulsified compounds were stored at 4 °C for 6 months. Group A–E was injected into 5 pigs, respectively, and the other 5 were injected with newly emulsified agent. The dose of emulsion injected was 2 mL. Group F was used as the control without sustained-release agent. Group G was used as the negative control without sustained-release agent and CpG. A summary of the experimental design is shown in Table 1. The piglets were vaccinated intramuscularly (i.m.) in the neck at the same time with vaccine.

### 2.3. Temperature Measurement

The rectal temperature of piglets in each group was measured daily on the first 7 days after all pigs vaccinated with CSF live vaccine at about 10:00 a.m. Meanwhile, the allergic reactions at the injection site, such as swelling and absorption caused by injection, were observed. The pigs should be as quiet as possible when measuring the body temperature using forehead thermometer.

### 2.4. Relative Daily Weight Gain

The change in all pigs’ body weight was recorded at the time before vaccine (dpv 0) and 60 days later (dpv 60). Group average relative daily gain (the sum of IRDG/10) was calculated to evaluate the vaccine’s effects.

Individual relative daily gain (IRDG) = (dpv 60 weight − dpv 0 weight)/dpv 0 weight/60

Group average relative daily gain (GARDG) = The sum of IRDG/10

### 2.5. Assay of Pig Blood Antibody Levels and Cell Immunity

Blood samples were collected on 0, 7, 14, 21, 60 days after vaccination, centrifuged at 3000 g for 10 min at 4 °C, and the serum was separated and stored at −80 °C until testing. CSFV antibody levels were detected with CSFV ELISA kit (IDEXX) according to the manufacturer’s directions. The OD450 (ODTEST), ODPOS (positive control), ODNEG (negative control) was detected and blocking rate was calculated.

Blocking rate = (ODNEG − ODTEST)/ODNEG × 100%.

Vaccine cell immunity status was detected with pig inter-leukin-4 (IL-4) ELISA kit (CUSABIO) according to the manufacturer’s directions. The absorbance of each well was read in a spectrophotometer at 450 nm. Serial dilutions of standard samples were used to quantify the contents, and served as a standard curve. Then IL-4 contents in the serum samples were calculated.

### 2.6. Statistical Analysis

All data was analyzed and presented using Graphpad Prism 5 software. One-way analysis of variance (ANOVA) was used to do the statistical analyses followed by Tukey’s post hoc test, respectively. Differences were considered significant at *p* < 0.05.

## 3. Results

### 3.1. Temperature Measurement

The rectal temperature of all piglets on the first 7 days after vaccination was tested. The results showed that the temperature of all pigs fluctuated, but in no cases exceeded 40.5 °C for three consecutive days, indicating that the injection did not cause a fever reaction. There were no allergic reactions such as redness, swelling, heat or pain at the injection site, and no significant changes in the spirit and appetite of all pigs. This proves that the vaccine and the selected immune enhancer, emulsified with different sustained-release agents, is safe for piglets. At the same time, there was no significant difference between the emulsified infection stored for six months with the new emulsion.

### 3.2. Relative Daily Weight Gain

**Figure 1:**
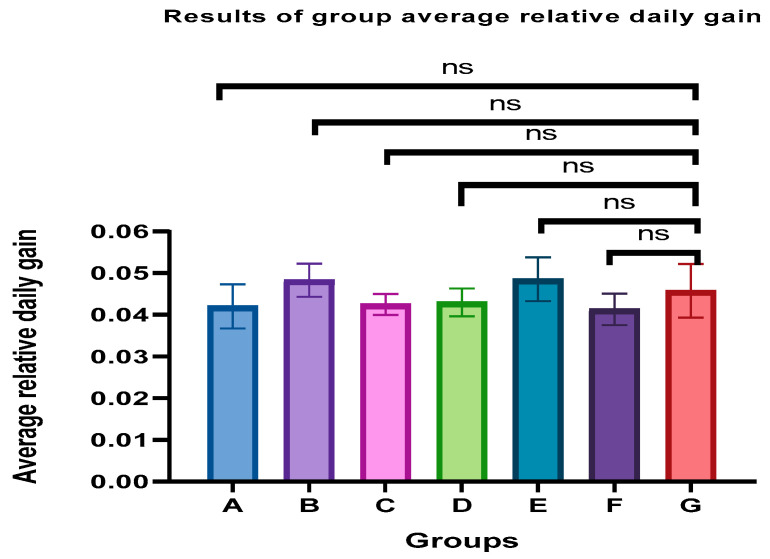
The results of group average relative daily gain. Compared to the control piglets (groups G), there was no significant difference among groups (*p* > 0.05). ns: no significant difference.

The *p*-value based on the GARDG of all immunoenhanced piglets was calculated and the results are shown in Figure 1. Compared to the control piglets (groups G), there was no significant difference in the immunoenhanced group (*p* > 0.05). The weight gain was not altered by vaccination. Also, the results showed that the emulsion stored for six months has no adverse effect on weight gain.

### 3.3. Serology

**Figure 2:**
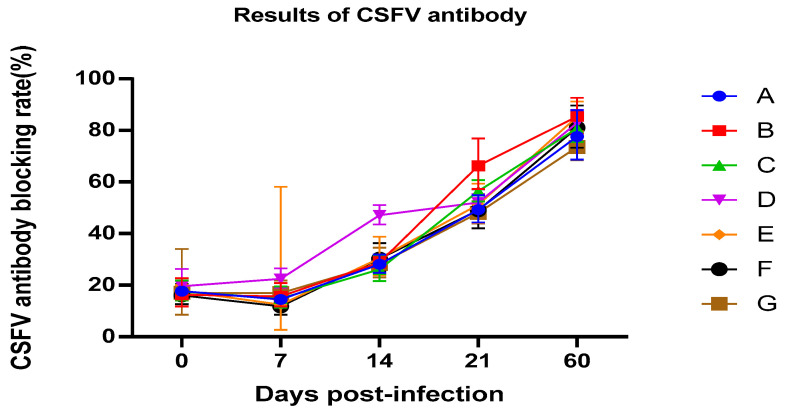
The results of classical swine fever virus (CSFV) blocking rate of all piglets detected with CSFV ELISA kit (IDEXX).

The blocking rate of all piglets is shown in Figure 2. It can be seen from the results that all experimental pigs can quickly produce swine fever antibodies after vaccination. On the 14th day after immunization, sustained-release agents used in group D can stimulate the rapid production of higher titers antibodies in experimental pigs. This result has important significance for emergency preventive immunity when swine fever occurs during production [12]. On the 21st day after immunization, the antibodies in the group injected with CpG and sustained-release agents were higher than those in the group injected with the vaccine alone, indicating that the CpG had shown good immune enhancement effects. Among the groups injected with emulsion, group B showed the highest antibody titer. On the 60th day after immunization, the antibody titers of all groups treated with emulsion were higher than those in group G. At the same time, there was no significant difference between the group vaccinated with the emulsion stored for 6 months and the new emulsion at the testing time point. This result showed that the emulsion stored for six months does not affect the effectiveness.

The antibody of group D is higher than other groups on the 14th day after immunization; group B showed the highest antibody titer on the 21st day after immunization. The antibody titers of groups with the immunoenhanced CpG and vaccine were higher than group G, who received only the vaccine, at 60 days post infection.

### 3.4. Changes in IL-4 Content in Piglets

IL-4 has been called the “prototypic immunoregulatory cytokine” [13]. Like many cytokines, it can affect a variety of target cells in multiple ways. IL-4 has an important role in regulating antibody production, hematopoiesis and inflammation, and the development of effector T-cell responses. Therefore, IL-4 can be used as an important indicator of cellular immunity.

**Figure 3:**
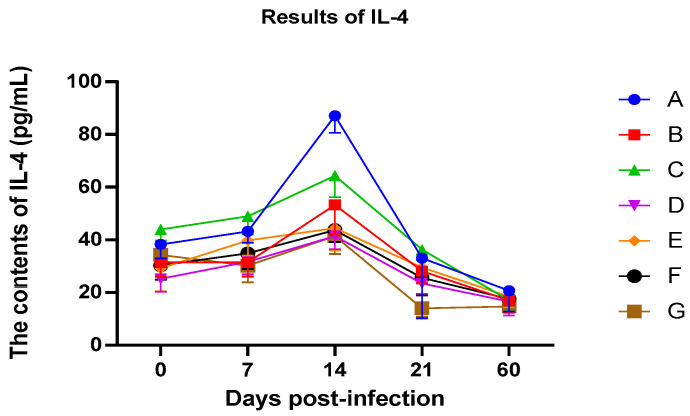
Level of interleukin-4 (IL-4) content in peripheral blood.

The IL-4 contents in the serum samples were calculated after serial dilutions of standard samples were used to serve as a standard curve and the results are shown in Figure 3. After vaccination, the levels of IL-4 in all pigs increased, indicating that the pigs produced a cellular immune response. On the 14th day after immunization, the IL-4 concentration reaches its peak.

The groups A–F IL-4 levels were higher than those in group G. These results indicated that CpG and sustained-release agents can better stimulate the pigs to produce cellular immune responses. The A–C group, especially group A and C, showed significant effects with stronger cellular immunity. After 14 days, the IL-4 contents began to gradually decrease, but groups A–F were still higher than group G, indicating that the emulsion used still stimulated the pigs to produce cellular immune responses for a long time. On the 60th day after immunization, all groups approached normal levels. It is speculated that the reason for this being lower than before immunization was that immune and other stimulating factors can also affect the body’s IL-4 levels.

On the 14th day after immunization, the IL-4 concentration reached its peak. The levels in groups A–F were higher than those in group G. After 14 days, the IL-4 contents began to gradually decrease, but groups A–F were still higher than group G.

## 4. Discussion

Immune adjuvants are now widely used in combination with various vaccines. In addition to enhancing the immune response, they also have the advantages of reducing antigen usage, efficiently activating the immune system, and prolonging the titer level. They have important value for enhancing the vaccine immune effect. Now they are an indispensable focus material in global vaccine research and development.

At present, aluminum adjuvant is still the most widely approved vaccine adjuvant used in China. Aluminum adjuvants are usually effective in increasing serum antibodies, but they can cause injection site reactions and weak ability to induce cellular immunity, which limits their application and does not have significant effects on certain vaccines. Nowadays, the direction of vaccine research is expanding and extending from preventing infectious diseases to treating chronic infectious diseases, cancer, and immune system diseases, which undoubtedly brings new challenges and opportunities for the development of new adjuvants.

CpG-ODN adjuvant can not only induce specific immune cell differentiation and proliferation, and enhance the immunogenicity of the vaccine, but also enhance antigen-specific humoral immunity in the vaccine development process [11]. However, due to the presence of nucleases, CPG is easily degraded, and sustained-release agents are necessary for its long-term effect in immune enhancement and immune regulation in the body. The sustained-release agents used in this study are commonly commercialized materials, which will not significantly increase the cost of vaccination. PEG classified as a non-immunogenic polymer has been applicated in many other products, such as cosmetics, foods and pharmaceutical preparations [12]. PEG can also coat vaccine products to maintain their stability [14].

As a kind of antibacterial and antifungal preservative, benzoic acid is widely used in foods and feeds. Recently, many studies showed that using appropriate benzoic acid can regulate enzyme activity, redox status, immunity and microbiota, which can also enhance the immunogenicity of vaccine antigens [15]. As a widely used adjuvant in inactivated vaccines, white oil can delayed the retention time of immunogens, allow them to be continuously and slowly released, and enhance the phagocytic and bactericidal abilities of macrophages [16].

Through comparative experiment, it was found that the vaccine combined with immune enhancers and sustained-release agents has no safety issues. The application effect showed that sustained-release agents PEG20000, benzoic acid and Seppic white oil have more significant effects on improving humoral immunity, and total white oil, PEG6000, benzoic acid and Seppic white oil have better effects on cellular immunity. In clinical production, there is some emergency situations, such as the occurrence of disease cases after segregation, where other healthy populations need to quickly improve their cellular and humoral immune levels. This research result can be combined with vaccines to achieve better results. Due to the different types of viruses, further experimental verification is needed to evaluate the effect of other vaccines.

## 5. Conclusions

In summary, the combination of CpG immune enhancers and sustained-release agents synergistically augmented both humoral and cellular immune responses. Specifically, PEG20000 and benzoic acid combination, and Seppic white oil significantly enhanced humoral immunity, as evidenced by elevated neutralizing antibody titers. In contrast, total white oil, PEG6000 and benzoic acid combination exhibited superior efficacy in promoting cellular immunity, with increased IL-4 levels indicating Th2-biased immune activation. These findings highlight the differential roles of sustained-release agents in tailoring immune outcomes and provide a strategic framework for optimizing CSFV vaccine formulations through adjuvant synergy.

## Figures and Tables

**Table 1:** Experimental design.

Groups	Sustained-Release Agents Name	Immunoenhancer/Dose	Sustained-Release Agents and CpG Ratio
A	Total white oil	18 CpG/800 ug	2:1
B	Seppic white oil	18 CpG/800 ug	1:1
C	PEG6000 and benzoic acid	18 CpG/800 ug	0.05% benzoic acid and 0.5% PEG
D	PEG20000 and benzoic acid	18 CpG/800 ug	
E	Defatted milk powder and sucro	18 CpG/800 ug	1:1
F	-	18 CpG/800 ug	-
G	-	-	-

## Data Availability

The raw data supporting the conclusions of this article will be made available by the authors on request.
